# Detection of macroenzymes: establishing upper reference limits for eight enzymes after polyethylene glycol precipitation

**DOI:** 10.11613/BM.2023.010705

**Published:** 2022-12-15

**Authors:** Lieselot Dedeene, Marie Stockman, Sophie Steels, Pieter Vermeersch, Glynis Frans

**Affiliations:** Clinical Department of Laboratory Medicine, University Hospitals Leuven, Leuven, Belgium

**Keywords:** precipitation, enzymes, macroamylase, polyethylene glycol, reference value

## Abstract

**Introduction:**

The presence of macroenzymes in blood can cause diagnostic confusion. Therefore, confirming the presence of macroenzymes is important to reduce unnecessary (non-)invasive investigations. Polyethylene glycol (PEG) precipitation is a simple and fast first-line method for the detection of macroenzymes. However, there is no consensus on the upper reference limit for the PEG-precipitable activity (%PPA) of monomeric enzymes. The aim of this study was to verify a PEG precipitation protocol for the detection of macroenzymes in our laboratory by establishing upper reference limits (URLs) and determining imprecision for eight enzymes after PEG precipitation. In addition, we aimed to clinically verify the URLs using samples containing macroenzymes as identified by electrophoresis.

**Materials and methods:**

*Per* enzyme, at least 40 leftover blood samples from adult patients with either normal or increased enzyme activities were diluted 1:1 with 25% PEG 6000 and 1:1 with 0.9% NaCl. Mixtures were incubated for 10 min at 37°C and centrifuged. Supernatant enzyme activity was measured on Cobas c702 and the %PPA was calculated.

**Results:**

The following URLs were obtained: 26% PPA for amylase, 29% PPA for alkaline phosphatase (ALP), 61% PPA for alanine aminotransferase, 48% PPA for aspartate aminotransferase, 24% PPA for creatine kinase (CK), 55% PPA for gamma-glutamyltransferase, 65% PPA for lactate dehydrogenase, and 56% PPA for lipase. The within-lab imprecision was < 15%. Regarding the clinical verification, the two historical samples with proven macroCK showed a %PPA of 69% and 43%, respectively, and a sample with proven macroALP had a %PPA of 52%.

**Conclusion:**

In this study, URLs for monomeric enzyme activities after PEG precipitation for eight different enzymes were established. The URLs are suitable for clinical use, but are only partially in line with other studies. Therefore, our data highlight the importance of establishing laboratory-specific upper reference limits for %PPA to allow a correct interpretation.

## Introduction

Macroenzymes are high-molecular weight complexes formed by association of enzymes with other plasma components (immunoglobulins, lipoproteins) or through self-polymerization (*1*, *2*). They typically show an increased plasma activity due to reduced clearance of the high-molecular weight complex. This may cause diagnostic confusion (*1*-*3*). Routine enzyme measurements cannot distinguish macroenzymes from monomeric enzymes, which delays the recognition of macroenzymes. Moreover, macroenzymes are not frequently encountered and the prevalence varies from less than 0.1% to 3.5%, complicating matters further (*4*). Amylase and creatine kinase (CK) are the most common types of macroenzymes reported in literature (*1*, *3*, *5*, *6*). Other reported types of macroenzymes include alkaline phosphatase (ALP), alanine aminotransferase (ALT), aspartate aminotransferase (AST), gamma-glutamyltransferase (GGT), lactate dehydrogenase (LDH) and lipase (*1*, *3*, *5*, *6*). Nevertheless, as macroenzymes are mainly considered benign, confirming the presence of a macroenzyme is important to reduce unnecessary repeated examinations (both non-invasive and invasive) and possible therapeutic errors (*7*).

Polyethylene glycol (PEG) precipitation is considered an easy-to-use and inexpensive method to detect macroenzymes based on the differential solubility of the high molecular weight complexes. Other more expensive, labour-intensive and/or time-consuming tests include electrophoresis, ultrafiltration and gel filtration chromatography, all depending on the distinct size of macroenzymes. The latter three tests are often executed in specialized labs, thereby extending the turnaround time. For patients with higher (isolated) enzyme activities and no concordant clinical symptoms and/or imaging studies, PEG precipitation can be quickly indicative of the presence of macroenzymes when the PEG-precipitable activity (%PPA) is above the upper reference limit (URL).

However, one has to keep in mind the limitations of this first-line method. For example, high %PPA in patients with an excess of immunoglobulins should be interpreted with caution as monomeric enzymes might be precipitated along with the immunoglobulins, which has been demonstrated in patients infected with human immunodeficiency virus (*7*, *8*). Thus, when in doubt of a possible false-positive or false-negative PEG precipitation result, more sensitive and specific techniques (*e.g.*, electrophoresis and gel filtration) or the determination of urinary clearance ratios should be considered. This emphasizes the importance of primarily testing patients that are highly suspected for the presence of a macroenzyme based on initial clinical and technical examinations.

To date, only limited studies are available establishing reference intervals for the %PPA of monomeric enzymes (*1*, *3*, *5*). Furthermore, established reference intervals vary widely between different enzymes and seem to be lab-dependent, which can be partially explained by the assay used (including calibrator traceability), variation in PEG precipitation protocols, and the type of PEG used. This hampers the transferability of reference intervals between laboratories and, consequently, the correct interpretation of %PPA for a particular enzyme or single sample. In addition, the modification of a CE-IVD labelled method to identify macroenzymes requires validation as a lab-developed test after May 2022 according to the European Union In Vitro Diagnostic Regulation 2017/746 (*9*). The aim of the study was to verify an accessible and fast first-line method for the detection of macroenzymes in suspected patients in our laboratory by establishing upper reference limits (URLs) and determining imprecision for eight enzymes after PEG precipitation. In addition, we aimed to clinically verify the URLs using samples containing macroenzymes as identified by electrophoresis.

## Materials and methods

### Subjects

The study was approved by the Ethics Committee at University Hospitals Leuven (Study number S66607). To establish URLs for the % PEG-precipitable activity (%PPA), at least 40 randomly selected leftover blood samples from adult patients with either normal or increased enzyme activities were included *per* enzyme. For the imprecision study, the plasma pool was derived from 10 leftover blood samples from adult patients. The %PPA was determined for each enzyme on the plasma pool before starting the imprecision experiments. If the %PPA was above the URL for a specific enzyme, a new plasma pool was made and used for this enzyme. To perform a clinical verification of the URL, the %PPA of three historical samples with macroenzymes confirmed by electrophoresis was determined (macroCK: N = 2, macroALP: N = 1). These historical samples were stored at -20°C until use. To verify the absence of artificial precipitation effects caused by storage conditions, three control samples with elevated enzyme activities and a similar storage time (± 1 year for samples ≥ 1 years old or ± 1 month for samples < 1 years old), but without the presence of macroenzymes, were analysed simultaneously. This study used the leftover samples from the clinical laboratory and complied with all national regulations, institutional policies and was performed in accordance with the Helsinki Declaration.

### Methods

**Figure 1:**
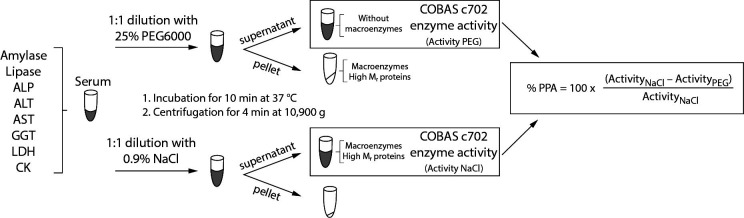
PEG precipitation protocol and calculation of %PPA. %PPA – percent PEG-precipitable activity. PEG - polyethylene glycol. ALP - alkaline phosphatase. ALT - alanine aminotransferase. AST - aspartate aminotransferase. CK - creatine kinase. GGT – gamma-glutamyltransferase. LDH - lactate dehydrogenase.

The protocol for PEG precipitation was adapted from a previously published protocol (*2*) (Figure 1). Samples were diluted 1:1 with 25% PEG 6000 and 1:1 with 0.9% sodium chloride (NaCl). Mixtures were incubated for 10 minutes at 37°C and centrifuged for 5 minutes at 10,900xg. Afterwards, supernatant enzyme activity was measured on Cobas 8000 c702 (Roche Diagnostics, Basel, Switzerland) (Table 1) and the %PPA was calculated with the following formula: %PPA = 100 x [(Activity_NaCl_ – Activity_PEG_)/Activity_NaCl_] (10-21). All c702 assays were performed according to the manufacturer’s instructions.

**Table 1:** Analytical specifications and reference values of the enzymes

**Enzyme**	**Test principle**	**Calibrator traceability**	**Reference values (≥ 18 years)**		
			**Male**	**Female**	**Reference**
Amylase	Colorimetric assay – IFCC method	Roche reagent according to IFCC (10)	28-100 U/L	28-100 U/L	(*10*,*11*)
ALP	Colorimetric assay – IFCC method	IFCC formulation (12)	40-130 U/L	35-105 U/L	(*12*)
ALT	Colorimetric assay – IFCC method without pyridoxal phosphate activation	IFCC formulation (14)	≤ 41 U/L	≤ 31 U/L	(*14*,*15*)
AST	Colorimetric assay – IFCC method without pyridoxal phosphate activation	IFCC formulation (16)	≤ 37 U/L	≤ 31 U/L	(*16*)
CK	Colorimetric assay – IFCC method	IFCC formulation (17)	≤ 190 U/L	≤ 170 U/L	(*13*,*17*)
GGT	Colorimetric assay – IFCC method	IFCC formulation (18)	≤ 60 U/L	≤ 40 U/L	(*13*,*18*)
LDH	UV assay – IFCC method	IFCC formulation (19)	135-250 U/L^†^	135-250 U/L^†^	(*19*,*20*)
Lipase	Enzymatic colorimetric assay	Roche reagent	13-60 U/L	13-60 U/L	(*21*)
All analyses were performed on the Cobas c702 analyzer. ^†^For adult patients ≥ 19 years. ALP - alkaline phosphatase. ALT - alanine aminotransferase. AST - aspartate aminotransferase. CK - creatine kinase. GGT - gamma-glutamyltransferase. LDH - lactate dehydrogenase. UV – ultraviolet. IFCC - The International Federation of Clinical Chemistry and Laboratory Medicine.					

### Statistical analysis

The within-lab imprecision of the plasma pool (five measurements *per* day for five days) was determined by one-way analysis of variance (ANOVA) according to the CLSI EP15-A3 guideline (*22*). Normality of the data was analysed using the Shapiro-Wilk test. The URLs for %PPA (including the 95% confidence intervals) were calculated for each enzyme using the nonparametric method with bootstrap resampling (97.5^th^ percentile, 500 times resampled) after outlier exclusion with the Dixon’s test (*23*, *24*). Spearman’s test was used to explore correlations for all enzymes between (i) %PPA and age and (ii) %PPA and the initial enzyme activity. A Mann-Whitney U test was performed to assess the difference in %PPA between men and women for all enzymes. The significance level was set at 0.01 to account for multiple testing. Statistical analysis was performed with GraphPad Prism (version 9.2.0, Graphpad Software, San Diego, USA), Analyse-it Method validation edition (version 5.65.3, Analyse-it Software, Leeds, UK), and Excel with the Analysis Toolpak (Microsoft, version 2207, Redmond, USA).

## Results

**Figure 2:**
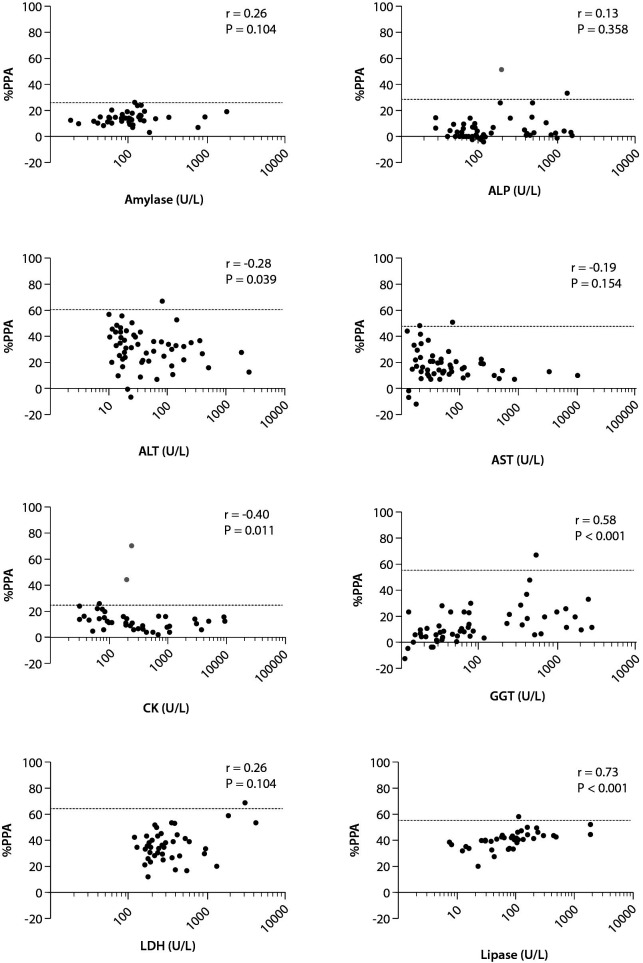
Spearman’s correlation analysis between the initial enzyme activity and %PPA for all enzymes. Spearman’s correlation coefficient r and associated P values are shown on the graphs. The significance level was set at α = 0.01 to account for multiple testing. The URL is represented by a dashed line. Grey dots represent the %PPA and initial enzyme concentration of samples with macroenzymes (N=3), which were not included in the statistical analysis. %PPA – percent PEG-precipitable activity. ALP - alkaline phosphatase. ALT - alanine aminotransferase. AST - aspartate aminotransferase. CK - creatine kinase. GGT – gamma-glutamyltransferase. LDH - lactate dehydrogenase.

Table 2 describes the established URL and within-lab imprecision for the eight investigated enzymes. The within-lab imprecision was < 15%. The number of samples, median and range of the enzyme activity and demographics of the study population are also presented in Table 2. No significant correlation was observed between %PPA and age of the patients for all enzymes (Table 3). No significant difference in %PPA was observed between men and women for all enzymes (Table 3). A significant positive correlation (P < 0.001) was observed between %PPA and the initial enzyme activity for GGT and lipase, but not for the other enzymes (Figure 2). Regarding the clinical verification, the two historical samples with proven macroCK showed a %PPA of 69% and 43%, respectively, which is above the URL of 24% (Figure 2). The sample with proven macroALP had a %PPA of 52%, which is above the URL of 29% (Figure 2). The simultaneously analysed control samples had %PPA levels below the URL (for CK: 15 and – 13%PPA, and for ALP: 12%PPA).

**Table 2:** Upper reference limits and within-lab imprecision for eight enzymes after PEG precipitation

	**Upper reference limit (%PPA)**				Imprecision PEG precipitation*	
**Enzyme**	**N samples** **after outlier exclusion** **(male:female)**	**Patients age, years**	**Initial activity, U/L (median and IQR)^†^**	**URL, %PPA (95% CI)**	**Initial activity plasma pool (U/L)**	**Within-lab imprecision (%)**
Amylase	41 (27:14)	64 (31-90)	106 (61-143)	26 (24-26)	114	3.1
ALP	55 (33:22)	62 (29-90)	93 (64-405)	29 (15-34)	442	2.9
ALT	54 (34:20)	62 (29-90)	30 (17-108)	61 (52-67)	204	4.9
AST	55 (35:20)	62 (29-90)	44 (22-113)	48 (40-51)	254	2.4
CK	39 (29:10)	56 (29-90)	227 (80-921)	24 (21-25)	322	3.3
GGT	54 (34:20)	62 (29-90)	62 (29-417)	55 (32-67)	322	2.5
LDH	42 (30:12)	62 (29-90)	264 (189-426)	65 (54-69)	454	2.5
Lipase	40 (26:14)	62 (29-90)	86 (33-151)	56 (50-58)	261	10.6
*Evaluated on absolute enzyme activities after PEG precipitation. ^†^Reference intervals for enzyme activities are shown in Table 1. Age is presented as median (range). %PPA – percent PEG-precipitable activity. IQR - interquartile range. PEG - polyethylene glycol. URL - upper reference limit. ALP - alkaline phosphatase. ALT - alanine aminotransferase. AST - aspartate aminotransferase. CK - creatine kinase. GGT - gamma-glutamyltransferase. LDH - lactate dehydrogenase.						

**Table 3:** Mann-Whitney U test for the difference in %PPA between men and women and correlation analysis of %PPA and age

**Enzyme**	**Median %PPA of male patients**	**Median %PPA of female patients**	**P**	**Spearman correlation coefficient between age and %PPA (P-value)**
Amylase	14	14	0.518	0.09 (0.589)
ALP	1	3	0.050	- 0.07 (0.631)
ALT	34	27	0.081	0.10 (0.484)
AST	17	15	0.658	0.19 (0.173)
CK	11	12	0.721	0.34 (0.032)
GGT	7	12	0.014	- 0.01 (0.974)
LDH	36	34	0.666	- 0.05 (0.738)
Lipase	40	42	0.834	- 0.01 (0.956)
The difference between male and female %PPA was tested using the Mann-Whitney U test. P < 0.01 was considered statistically significant. %PPA – percent PEG-precipitable activity. IQR - interquartile range. PEG - polyethylene glycol. URL - upper reference limit. ALP - alkaline phosphatase. ALT - alanine aminotransferase. AST - aspartate aminotransferase. CK - creatine kinase. GGT - gamma-glutamyltransferase. LDH - lactate dehydrogenase.				

## Discussion

In this study, we determined URLs for eight enzymes after PEG precipitation to detect the presence of macroenzymes. In addition, we demonstrated that the within-lab precision for the eight enzymes after PEG precipitation was adequate (imprecision < 15% for all enzymes). Furthermore, the newly established URLs for CK and ALP were clinically verified using three samples containing macroenzymes as identified by electrophoresis. These samples showed a %PPA higher than the URL for the respective enzyme.

Our study is not the first one to establish reference intervals after PEG precipitation but, to our knowledge, it is the first one to determine them for eight commonly measured enzymes in the clinical laboratory at the same time (*1*, *3*, *5*). The URLs established in the current study are in line with a previous study establishing reference intervals using samples with elevated enzyme activities (except for amylase) (*1*). In contrast, our URLs are considerably lower (except for GGT) as compared to the reference intervals established in a population of apparently healthy subjects without elevated enzyme activities (*3*, *5*). These differences might be explained by small variations in the PEG precipitation protocol (*e.g.,* incubation temperature, centrifugation time and speed, type of PEG). Furthermore, a different patient population with respect to the initial enzyme activity (*e.g.,* normal enzyme activities versus a mix of normal and elevated enzyme activities) might also contribute to variations in references values. However, the latter might only play a minor role, as our results showed that the %PPA did not depend on the initial enzyme activities for most of the enzymes (except for GGT and lipase for which the correlation coefficient revealed a moderate to good correlation). Furthermore, variations in reference values could be introduced by the analytical method itself. However, the previously mentioned studies and our study all use analytical assays from Roche Diagnostics, which lowers the chance that differences are majorly caused by the analytical method itself provided that the Roche assay methods and calibrator traceability did not considerably change over the years (*1*, *3*, *5*). Nevertheless, it remains unclear whether our URLs can be transferred to different assays (*e.g*. not traceable to IFCC methods and standard formulations), even if the same PEG precipitation protocol would be used. Finally, a limitation of our study is the limited number of samples used to determine the URLs, which might contribute to the observed differences as well.

In conclusion, we performed a method verification for PEG precipitation on Cobas c702 and established URLs for monomeric enzyme activities after PEG precipitation for eight different enzymes. The established URLs are suitable for clinical use, as verified by three samples containing macroenzymes. However, the URLs established in this study are only partially in line with previously published reference values. Therefore, our data highlight the importance of establishing enzyme-specific and laboratory-specific upper reference limits for %PPA to allow a correct interpretation. Future analysis of samples with and without macroenzymes will further validate the clinical utility of this PEG precipitation protocol in our laboratory.
